# Granulosa cell endothelin-2 expression is fundamental for ovulatory follicle rupture

**DOI:** 10.1038/s41598-017-00943-w

**Published:** 2017-04-11

**Authors:** Joseph A. Cacioppo, Po-Ching Patrick Lin, Patrick R. Hannon, Daniel R. McDougle, Arnon Gal, CheMyong Ko

**Affiliations:** 1 Department of Comparative Biosciences, College of Veterinary Medicine, University of Illinois at Urbana-Champaign, Urbana, IL 61802 USA; 2 Department of Obstetrics & Gynecology, University of Kentucky, Lexington, KY 40536 USA; 3 Department of Small Animal Internal Medicine, Institute of Veterinary, Animal and Biomedical Sciences, Massey University, Palmerston North, 4442 New Zealand

## Abstract

Ovulation is dependent upon numerous factors mediating follicular growth, vascularization, and ultimately oocyte release via follicle rupture. Endothelin-2 (EDN2) is a potent vasoconstrictor that is transiently produced prior to follicle rupture by granulosa cells of periovulatory follicles and induces ovarian contraction. To determine the role of *Edn2* expression, surgical transplant and novel conditional knockout mice were super-ovulated and analyzed. Conditional knockout mice utilized a new iCre driven by the *Esr2* promoter to selectively remove *Edn2*. Follicle rupture and fertility were significantly impaired in the absence of ovarian *Edn2* expression. When ovaries of Edn2KO mice were transplanted in wild type recipients, significantly more corpora lutea containing un-ovulated oocytes were present after hormonal stimulation (1.0 vs. 5.4, p = 0.010). Following selective ablation of *Edn2* in granulosa cells, Esr2-Edn2KO dams had reduced oocytes ovulated (3.8 vs. 16.4 oocytes/ovary) and smaller litters (4.29 ± l.02 vs. 8.50 pups/dam). However, the number of pregnancies per pairing was not different and the reproductive axis remained intact. Esr2-Edn2KO ovaries had a higher percentage of antral follicles and fewer corpora lutea; follicles progressed to the antral stage but many were unable to rupture. Conditional loss of endothelin receptor A in granulosa cells also decreased ovulation but did not affect fecundity. These data demonstrate that EDN2-induced intraovarian contraction is a critical trigger of normal ovulation and subsequent fecundity.

## Introduction

Endothelin-2 (*Edn2*) encodes a short 21-amino acid peptide^1^. EDN2 is one of three similar isoforms in the body that act through two endothelin receptors (EDNRA and EDNRB)^2, 3^. *Edn2* is expressed in the gastrointestinal tract, prostate, skin, testes, and notably is known to be transiently expressed in the stimulated rodent and human ovary^4^ *. Edn2* mRNA is expressed in murine granulosa cells of mature ovarian follicles immediately prior to ovulation for approximately two hours^3, 5–7^. This period corresponds to ovulation and early corpus luteum (CL) formation in the mouse. When ovulation is induced through hormone injection, *Edn2* expression occurs from 11–12 hours after hCG injection while ovulation occurs at approximately 12 hours after injection^8, 9^. EDN2 has been shown to be important for ovulation and CL formation through pharmacological inhibition and global knockout mouse approaches^5, 6, 10^.

EDN2 assists in mediating ovulation by inducing follicular contraction for oocyte expulsion. Endothelin receptor A (EDNRA) is localized in the theca externa layer of the ovarian follicle and in the associated smooth muscle of arteries and arterioles in both rodents and humans^7, 11, 12^. Alternatively, endothelin receptor B (EDNRB) is expressed largely in endothelial cells^13^ where it acts to clear endothelins from circulation^14–17^. EDN2 signaling through EDNRA induces smooth muscle contraction resulting in oocyte expulsion^18, 19^, specifically at the apex of the follicle wall^18^ where fewer contractile cells are present^20–22^. Chemically-induced ovarian contraction has been documented in the human^23–25^, cat^26^, and rabbit^27–29^. Previous work by Bridges *et al*. using receptor antagonists support that EDNRA is the critical receptor involved in ovulation: EDN2-mediated contraction was reduced by a specific EDNRA antagonist but not by a specific EDNRB antagonist^3, 11^. Additionally, mice that conditionally lack EDNRB demonstrate no reproductive deficits and instead give birth to more pups per litter and produce more corpora lutea^30^. Most recently, EDN2-induced contraction and follicular rupture has been visualized through pharmacological antagonist treatment and intravital multiphoton microscopy by Migone *et al*.^18^.

The present study was undertaken to confirm and expand upon the significance of *Edn2* expression in folliculogenesis, ovulation, and overall fertility/fecundity. It extends previous studies on EDN2 in ovulation that utilized pharmacological inhibition and global knockout animals. The present study generates novel mouse models with ovarian-specific *Edn2* loss, characterizes their reproductive phenotypes, and quantifies the effect of EDN2 as a contractile agonist on ovaries *ex vivo*. We hypothesized that mice lacking *Edn2* in all or part of the ovary will have decreased oocytes ovulated and consequently smaller litter sizes, with fewer or no corresponding corpora lutea; these mice would maintain normal ovarian development and folliculogenesis until ovulation. Two novel mouse model systems were utilized for this purpose. The *Edn2* gene was ablated in the entire ovary and also specifically in the granulosa cells using the Cre/LoxP system^31^ in an Edn2-flox mouse line.

A mouse strain that globally lacks *Edn2* ^32^ was first used to examine the role of EDN2 in ovarian development and early follicular growth, and then in hormonally induced ovulation. EDN2 global knockout (Edn2KO) mice expire from starvation, hypothermia, and/or emphysema^33^, and have impaired ovulatory abilities as well as other defects^10^. Global *Edn2* loss is lethal to juveniles at approximately eight days of age, and organ transplant to healthy WT recipients was used to overcome this barrier to generate a mouse model that lacks *Edn2* in the ovary only. This ovary-specific *Edn2* ablation was achieved by transplanting ovaries of global Edn2KO to the kidney capsule of ovariectomized wild type (WT) mice to create a novel mouse model for examining the role of EDN2 in follicular development and ovulation.

Second, conditional *Edn2* ablation in the ovary was achieved by crossing floxed *Edn2* mice with mouse lines that express Cre recombinase driven by promoters for either progesterone receptor (*Pgr*, Pgr-Cre)^34^, aromatase (*Cyp19*; Cyp19-iCre)^35^, or estrogen receptor beta (*Esr2*, Esr2-iCre)^36–40^. These three conditional knockout models were generated to induce *Edn2* deletion in the granulosa cells at different stages of follicular development. Timing of genetic ablation via these Cre mice ranges from the primary follicle stage (Esr2-iCre)^37, 41–43^ to the secondary follicle stage (Cyp19-iCre)^44–46^, and to just six hours before ovulation (Pgr-Cre)^47, 48^, allowing selective removal of *Edn2* at different time points during follicular development and ovulation. Lastly, the significance of EDNRA-mediated contraction in ovulation was assessed by a selective ablation of *Ednra* in the ovarian cells and its impact on ovulation and ovarian contractility.

## Results

### Global Edn2KO mice have normal follicular development

**Figure 1:**
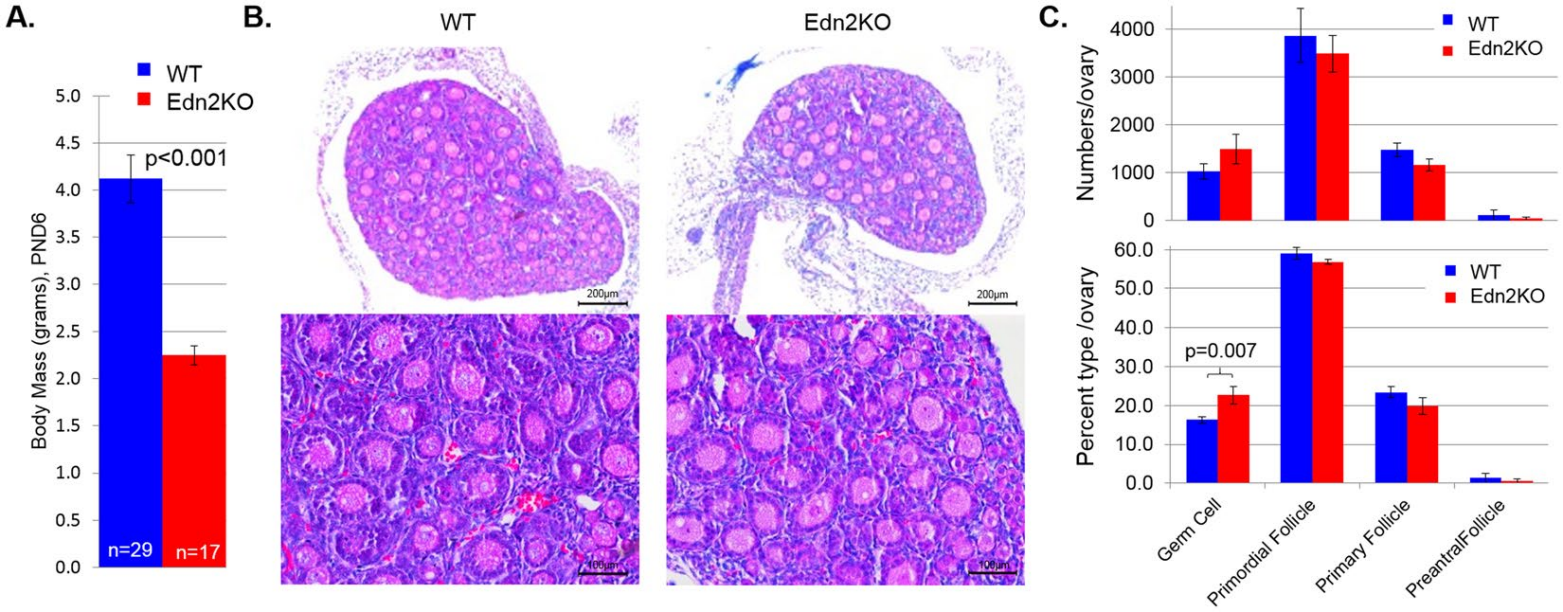
Edn2KO PND6 pups are smaller but have normal ovarian development. (**A**) Average body mass at post-natal day 6 (PND6) of WT (4.12 ± 0.25 g) vs Edn2KO (2.25 ± 0.10 g) pups. (**B**) Representative PND6 WT and Edn2KO ovaries sectioned at 5 μM. Histology images are shown at 10X and 20X. Both ovaries have a mix of germ cells, primordial follicles, primary follicles, and several preantral follicles. (**C**) Follicle and germ cell counting was performed on PND6 WT and Edn2KO and control ovaries (n = 10). Absolute cell counts are displayed above and the percentage of total cells per ovary is displayed below. A significant difference was seen between the percentage of germ cells present (p = 0.007). No other significant differences were noted. Error bars display the S.E.M.

Global *Edn2* knockout (Edn2KO) mice were generated by breeding floxed Edn2 mice (Edn2Flox) with Zp3-Cre mice, allowing Cre-mediated gene deletion at the oocyte stage^32^. The Edn2KO mice generated were comparable to previous reports on global *Edn2* loss by Chang *et al*.^33^: mice were unhealthy and generally expired by 8–10 days of age (range 0–18 days). Edn2KO mice were noticeably smaller by post-natal day 6 (PND6) despite heat/nutritional supplementation with Peptamen (Nestle), which suggests that observed growth retardation was due to starvation or early postnatal developmental defects (Fig. 1A). Although nearly half the size of their wild type (WT) siblings at PND6, ovarian/follicular development was largely unaffected. Ovaries examined at PND6 appear histologically normal (Fig. 1B) and had follicles of all expected levels of maturity. There was no difference between Edn2KO and WT ovaries in the absolute numbers of germ cells, primordial, primary, or preantral follicles per ovary (Fig. 1C). Additionally, there was no difference in the percentage of these follicles relative to total follicle plus germ cell number, except germ cell percentages were slightly elevated (p = 0.007) in Edn2KO PND6 ovaries. These data indicate that germ cell maturation to the primordial follicle stage proceeds normally to PND6 in the absence of EDN2.

### Ovulation is impaired in the Edn2-deficient ovary

**Figure 2:**
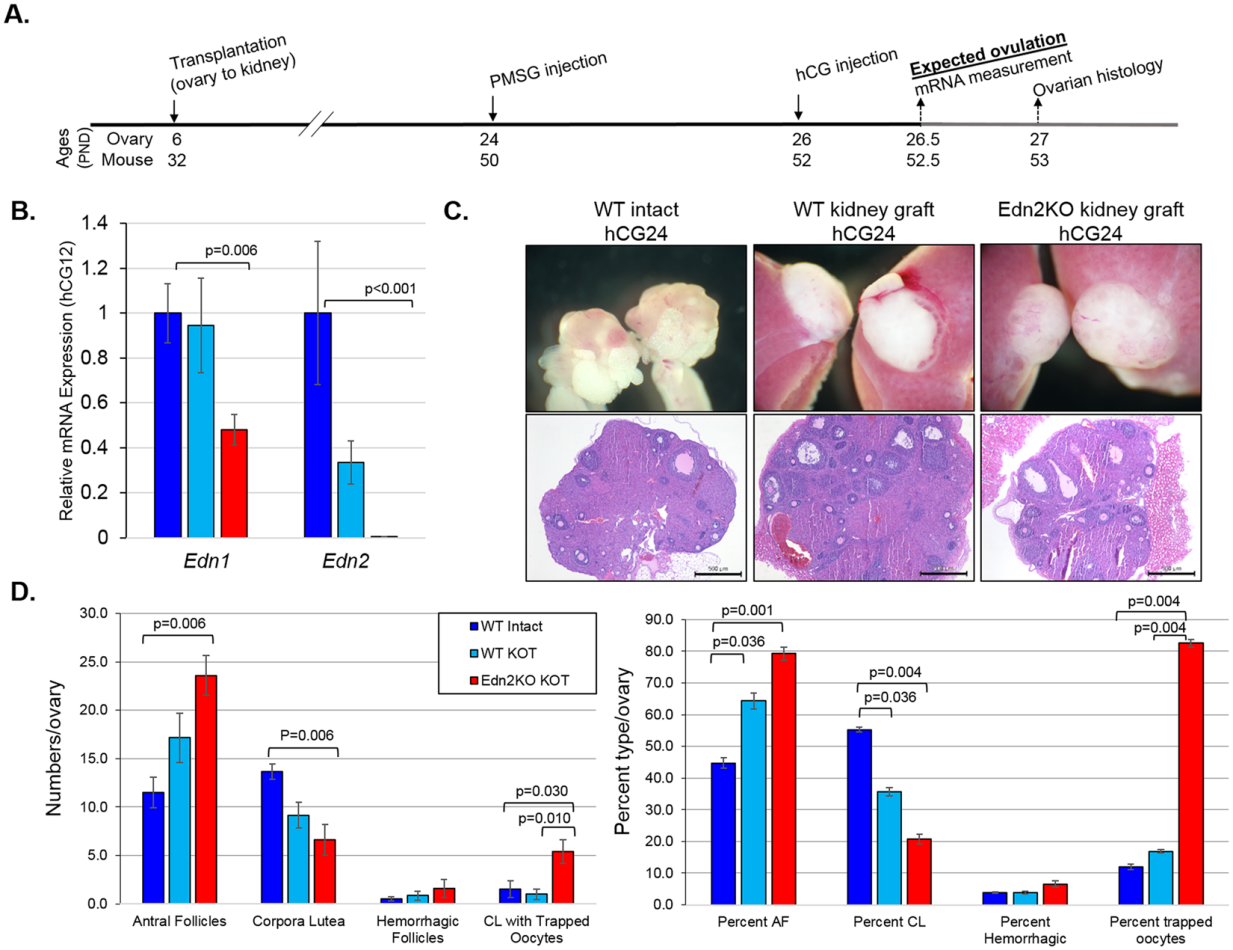
Edn2KO ovaries grafted under the kidney capsule have decreased ovulatory ability. (**A**) Experimental plan for WT and Edn2KO ovary grafting. Pups were euthanized at post-natal day 6 (PND6) and ovaries were grafted under the kidney capsule of 28–32 day old control females. Mice were then superovulated 18 days after transplantation and ovaries were collected. (**B**) Ovaries were collected at hCG12 hours; RNA was extracted and RT-PCR was performed for *Edn1* and *Edn2* expression to confirm gene loss. Grafted Edn2KO ovaries had significantly lower *Edn1* (p = 0.006) and *Edn2* (p < 0.001) expression than intact WT mice. Grafted WT ovaries trended towards decreased *Edn2* expression (p = 0.086). (**C**) At hCG 24 hrs, ovaries were collected and serially sectioned. Images are shown grossly before removal from the kidney and at 4X. Grafted ovaries become flattened with a greater diameter and are less deep. Antral follicles, corpora lutea, and immature follicles were present in all groups. (**D**) Ovaries collected at hCG 24 hrs were serially sectioned. The total numbers of antral follicles (AF) and corpora lutea (CL) were determined (n = 5–7). Absolute counts are displayed at left and percentages are displayed at right. When compared to grafted WT ovaries, transplanted Edn2KO ovaries trended towards a higher percentage of antral follicles and fewer CL (p = 0.088). Grafted Edn2KO ovaries had significantly more oocytes trapped within corpora luteal (p = 0.010) and a significantly greater percentage of CL with trapped oocytes (p = 0.004). Errors bars represent the SEM. KOT = kidney-ovarian transplant.

To determine the ovary-specific role of EDN2, Edn2KO ovaries and WT controls were removed at PND6 and grafted under the kidney capsule of ovariectomized (OVX) WT mice, generating Edn2KO Kidney-Ovary-Transplant (KOT) ovaries and WT KOT ovaries, respectively. This was employed to remove confounding systemic effects of *Edn2* loss. Ovaries were given time to mature to 24 days of age as response to gonadotropin stimulation is observed in WT intact mice by this age, and mice with surgical treatment had resumed normal estrous cycling by vaginal cytology at this time. At the grafted ovarian age of 24 days, ovulation was induced by injecting gonadotropins and ovaries and sera were collected at hCG 12 and hCG 24 hours (Fig. 2A). Quantitative RT-PCR was performed at hCG 12 hours to test that *Edn2* expression remained absent in Edn2KO KOT ovaries. Additionally, *Edn1* expression was examined due to its ability to signal via the same endothelin receptors with comparable affinity to EDN2^49^. Expression of *Edn2* was absent in Edn2KO KOT ovaries (red), indicating that ovarian EDN2 production did not occur after surgical graft nor after ovulation induction (Fig. 2B). Surprisingly, expression of *Edn1* was decreased (p = 0.006) in the grafted ovaries and compensatory expression did not occur. When histologically examined at hCG 24 hours, corpora lutea (CL) and antral follicles (AF) were present in both Edn2KO KOT ovaries and WT KOT ovaries (light blue), and WT intact age-matched mice (Fig. 2C). To quantitatively explore folliculogenesis, ovaries from hCG24 hours were serially sectioned and the numbers of follicles were compared among the groups. By absolute count per ovary, there were significantly more antral follicles (AF) but fewer CL in Edn2KO KOT ovaries than in WT intact ovaries (Fig. 2D, left). Similarly, there was significant difference in the percentage of AF and CL in Edn2KO KOT ovaries compared to WT (Fig. 2D, right). Importantly, Edn2KO KOT ovaries had a significantly increased percentage of oocytes trapped within CL compared to both WT intact and WT KOT ovaries (p = 0.004, 0.004, respectively). In Edn2KO ovaries, ovulation is impaired, but not completely abolished. Additionally, there was a greater percentage of AF and decreased percentage of CL in WT KOT ovaries compared to WT intact owing to surgical intervention. No differences in serum hormone concentrations of progesterone, 5β-corticosterone, or deoxycorticosterone (Figure S1) were observed at hCG12 or hCG24 hours. Taken together, these data demonstrate that ovulatory capacity is severely compromised in the Edn2KO ovary.

### Selective deletion of Edn2 in the granulosa cells impairs ovulation

**Figure 3:**
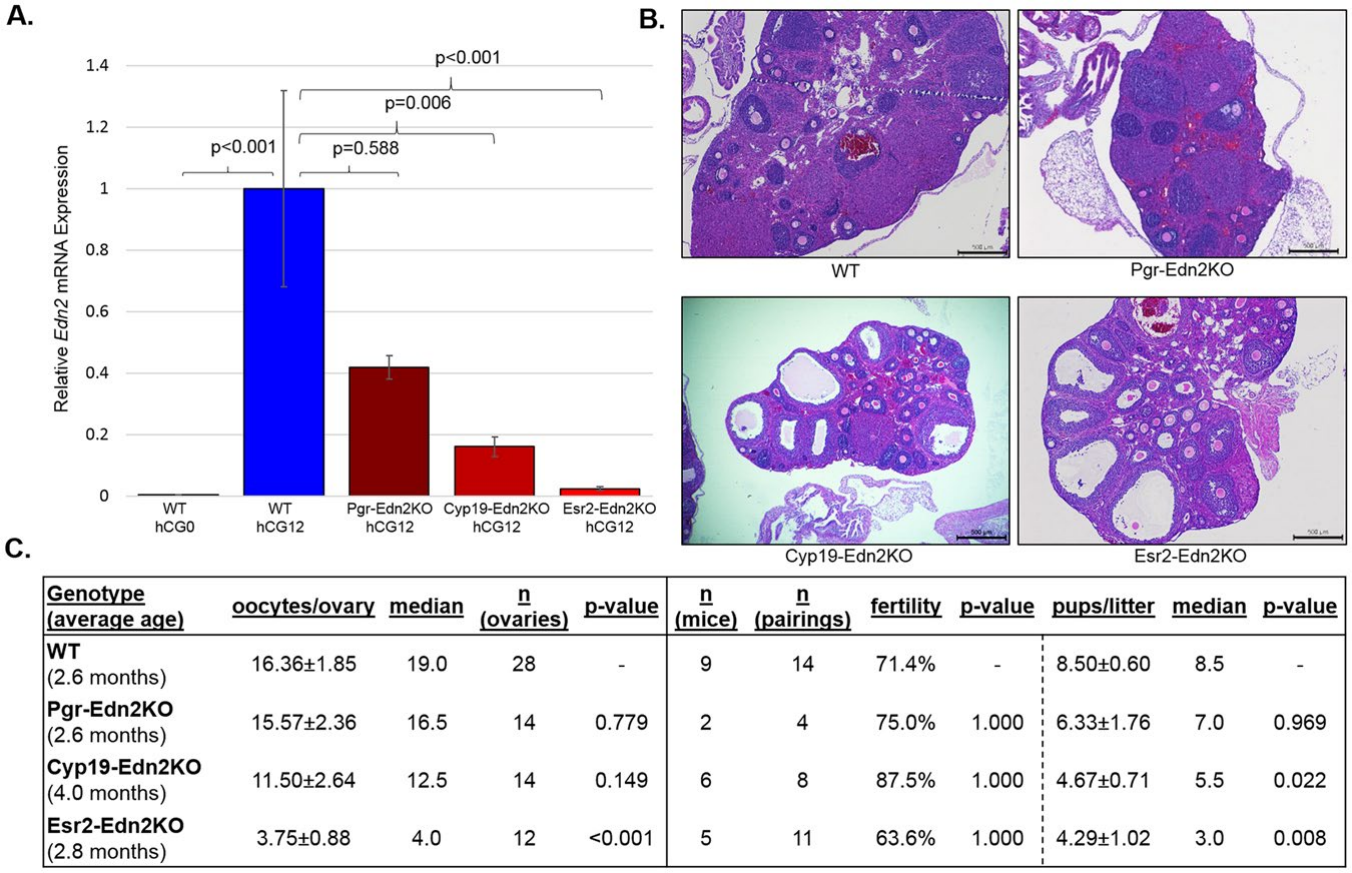
Loss of EDN2 reduces oocytes ovulated and decreases average litter size. Edn2flox mice were crossed with Pgr-Cre, Cyp19-iCre, and Esr2-iCre mice to remove *Edn2* expression in granulosa cells. (**A**) Relative *Edn2* mRNA expression in whole ovaries compared to WT hCG12 ovaries. Expression of *Edn2* is significantly lower in WT mice at hCG0 and in Cyp19-Edn2KO and Esr2-Edn2KO mice at hCG12 hours. (**B**) Representative histological images from ovaries of each genotype at hCG24 hours (4X). Ovaries from each genotype appeared healthy and all had corpora lutea, antral follicles, and immature follicles with no difference in hemorrhage between groups. Note the multiple antral follicles with trapped oocytes present in Cyp19-Edn2KO and Esr2-Edn2KO mouse lines. (**C**) Breeding study and superovulation oocyte collection results. Each pairing lasted 10 days; litter size represents pups present on day of birth; oocytes were collected from the ampulla of the oviduct at hCG24 hours. P-values are from the *post hoc* test relative to control mice. All error bars represent the SEM.

To overcome limitations of surgical transplantation, conditional *Edn2* knockout mice were generated. Three different mouse strains expressing Cre recombinase (or codon-improved Cre recombinase^50^, iCre) under the promoters of progesterone receptor (*Pgr*), aromatase (*Cyp19*), and estrogen receptor beta (ER-β; *Esr2*) were crossed with Edn2Flox mice. All mice expressed Cre in the granulosa cells. Expression of *Pgr* occurs in the granulosa cells of preovulatory follicles nine to six hours prior to ovulation in the mouse^47, 51^; *Cyp19* expression begins in the secondary follicles to allow estrogen production^52, 53^; and *Esr2* expression occurs within the granulosa cells of primary follicles following recruitment^40, 43, 54^. RT-PCR for *Edn2* at hCG12 revealed a graded decrease in expression relative to the time of Cre expression. Cyp19-Edn2KO and Esr2-Edn2KO mice had significantly reduced *Edn2* expression at hCG12 hours, while Pgr-Edn2KO mice had partially reduced mean *Edn2* expression that was not statistically significant (Fig. 3A). No changes in *Edn1* expression were observed. When examined histologically at hCG24, there was no difference in the number of hemorrhagic follicles, but Cyp19-Edn2KO and Esr2-Edn2KO ovaries had visibly more unruptured follicles present containing entrapped oocytes (Fig. 3B), similar to whole-ovary *Edn2* loss. Examination of the oviducts of ovulation-induced mice showed that Esr2-Edn2KO mice had significantly less oocytes than WT controls (3.75 ± 0.88 vs 16.36 ± 1.85 oocytes per ovary, p < 0.001), suggesting a defect in oocyte expulsion when *Edn2* is lost in the granulosa cells. To further probe this, a fertility assay with WT males revealed no significant difference in the percentage of litters born per pairing in any genotype (Fig. 3C). The average number of pups per litter was significantly reduced in Cyp19-Edn2KO and Esr2-Edn2KO mice compared to WT mice (4.67 ± 0.71 and 4.29 ± 1.02 vs. 8.50 ± 0.60, p = 0.022 and 0.008, respectively), directly associated to the difference in oocytes ovulated and *Edn2* expression. Pups were of normal weight at birth and matured normally. In particular, in the Esr2-Edn2KO mice gonadotropin stimulation resulted in ovulation of only four oocytes and the birth of four pups/litter on average, thus indicating that defects are likely exclusively in the ovulatory capacity in these mice. Pgr-Edn2KO mice displayed fertility and fecundity comparable to control mice and no significant ovulatory defect as well.

### Loss of EDN2 prevents transition from antral follicles to corpora lutea

**Figure 4:**
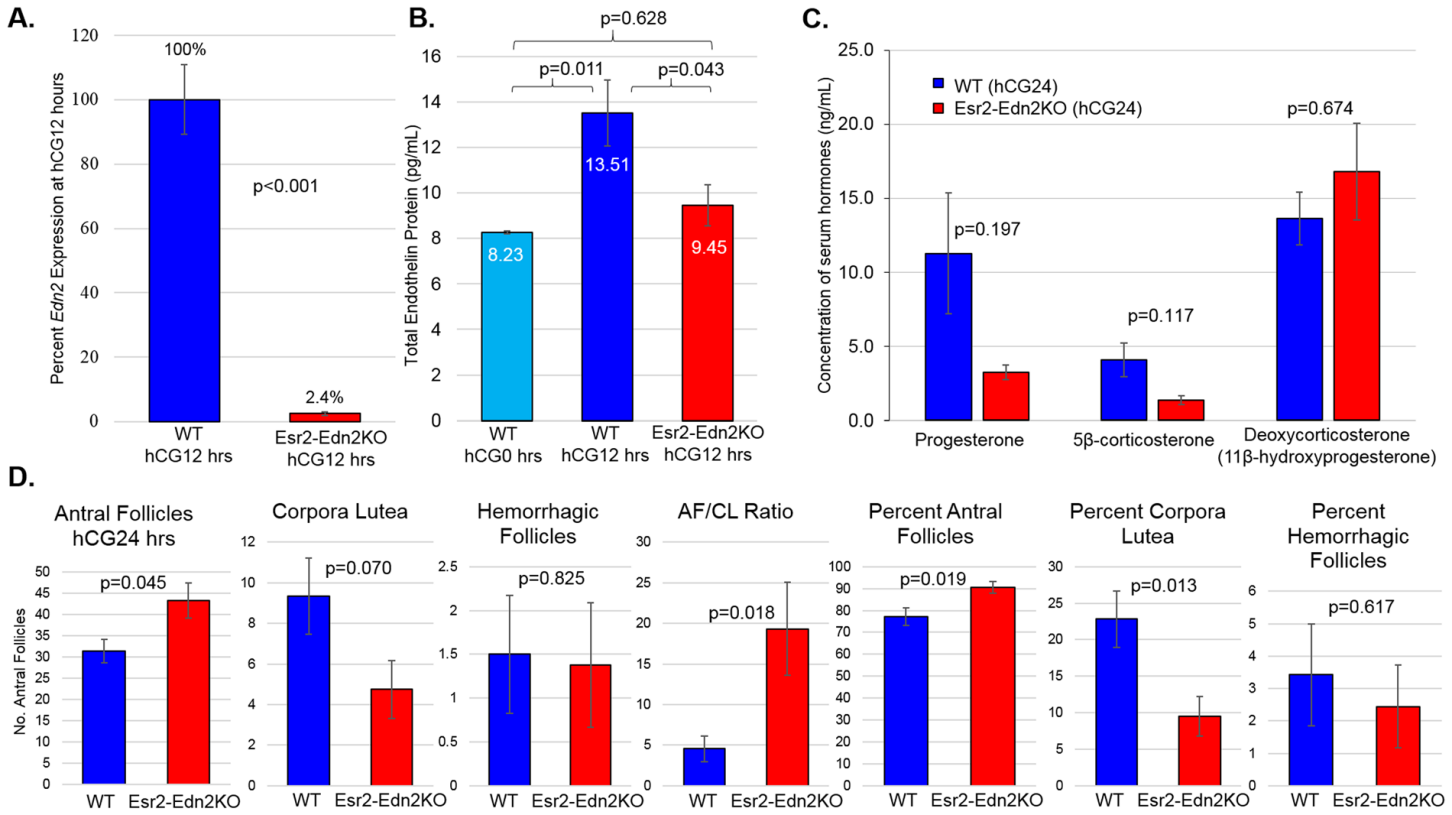
Esr2-Edn2KO mice have more antral follicles and fewer corpora lutea after ovulation. (**A**) Taqman quantitative RT-PCR results comparing *Edn2* expression in WT and Esr2-Edn2KO mice at hCG12 hours; 97.6% of *Edn2* expression is lost. (**B**) ELISA quantitation of total Endothelin protein per ovary. Endothelin mass is significantly reduced at hCG12 hours in Esr2-Edn2KO mice and is not different at hCG0 hours from WT mice. (**C**) Serum was extracted from PND25 mice at hCG24 hours and serum hormone concentrations (ng/mL) were quantified by LC/MS/MS. P-values represent ANOVA score by hormone. Error bars represent the SEM. (**D**) Ovaries were collected at hCG24 hours from PND25 mice, fixed, and serially sectioned. The absolute number of corpora lutea (CL), antral follicles (AF), and follicles with hemorrhage present were counted. The value is also expressed as a percentage of total ovarian structures. Error bars represent the SEM.

Esr2-Edn2KO mice showed the greatest reduction in *Edn2* expression among the conditional knockouts. *Edn2* was reduced by 97.6% percent in Esr2-Edn2KO mice (Fig. 4A). Correspondingly, endothelin protein expression was significantly reduced in whole ovaries at hCG12 hours (Fig. 4B). Total endothelin protein was measured by EIA; antibodies used were unable to discriminate between EDN1, EDN2, EDN3, and their prepro-forms. As *Edn1* and *Edn3* expression do not change in WT ovaries during ovulation^47^, the increase in endothelin peptide from hCG0 to hCG12 is presumed to be caused by an increase in *Edn2* transcription. Lack of *Edn2* upregulation thus accounts for the difference in endothelin protein content between WT and Esr2-Edn2KO mice at hCG12 hours. Multiple attempts at immunohistochemical localization of EDN2 using anti-EDN2 antibody (Abcam 197763)^55^ did not produce specific staining results (Figure S2). IHC staining was similar between WT, global Edn2KO, and granulosa cell-specific Edn2KO tissues and did not match phenotypic defects observed in global Edn2KO mice (expiration at eight days of age). As anti-Edn2 antibody was not specific for EDN2, it was not used to evaluate *Edn2* expression or EDN2 localization. Instead qRT-PCR and Immunometric ELISA quantification methods, which were consistent with each other as well as phenotypic data, were employed. The change in endothelin peptide produced a minor impact on serum progesterone concentration following ovulation at hCG24 hours (Fig. 4C). The trend towards decreased progesterone may be attributed to the lack of CL formed in Esr2-Edn2KO mice and high variability in WT measurements.

To quantify the differences in CL, or other ovarian structures present, follicle counting was performed on Esr2-Edn2KO ovaries from hCG24 hours after serial sectioning. Esr2-Edn2KO had significantly more antral follicles by total count and percentage, and a trend towards a lower percentage of CL (Fig. 4D, p = 0.07). The difference in antral follicles present (about 10AF) accounts for the difference in CL present (about 5CL) when considering that not all AF may form CL after ovulation. Based on the increased number of antral follicles entrapped oocytes, the fewer corpora lutea, and the increased number of trapped oocytes, loss of EDN2 results in reduced ovulation from an obvious defect in follicular rupture with subsequent lack of CL formation. Of note, pregnancy proceeds normally following ovulation in the Esr2-Edn2KO and luteinization occurs.

### Ednra ablation in granulosa cells has a minor impact on ovulation

**Figure 5:**
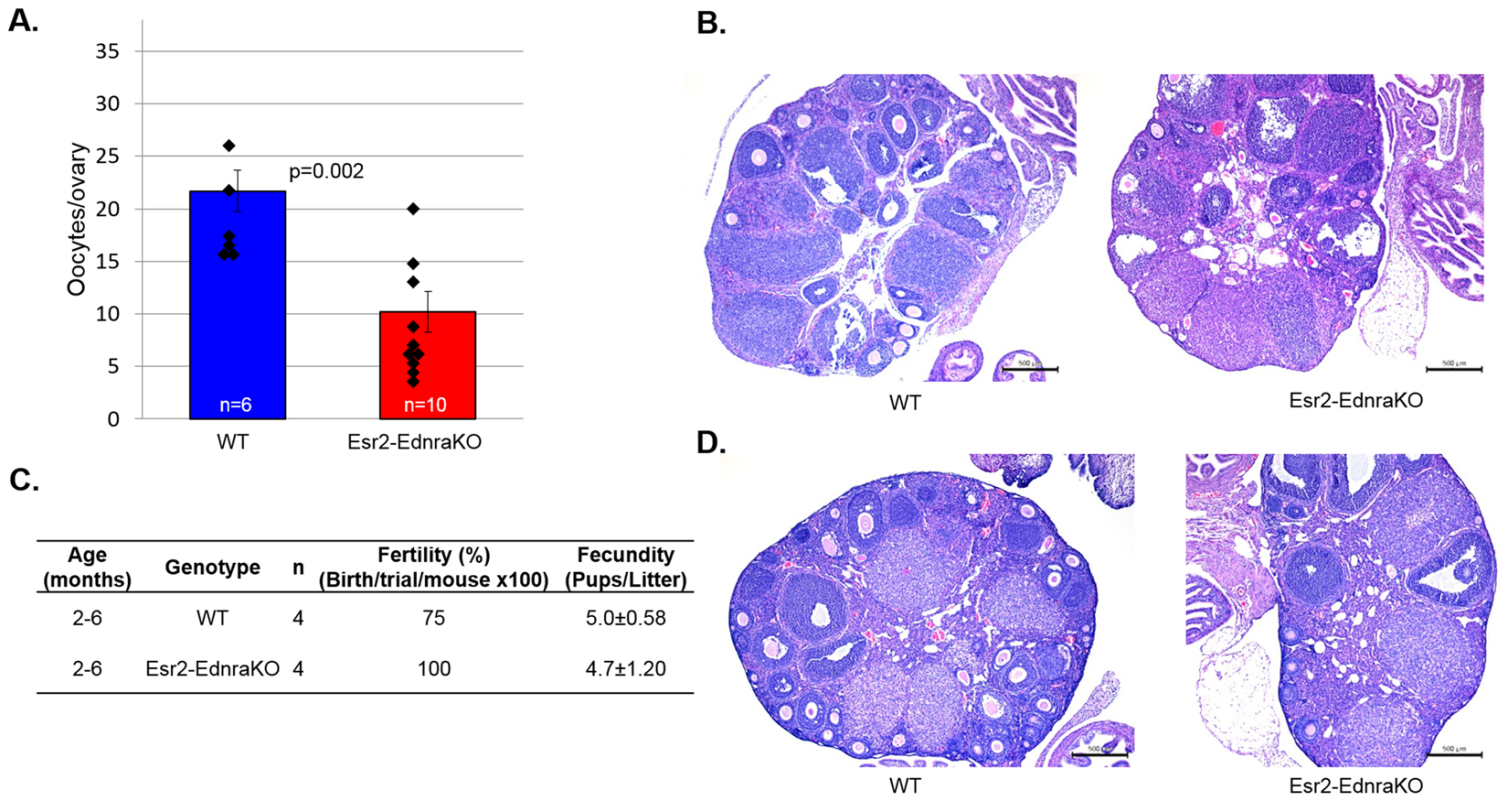
Ablation of Ednra in granulosa cells reduces ovulation. Ednra-flox mice were bred with Esr2-iCre mice to produce Esr2-EdnraKO mice. (**A** *)* After superovulation, oocytes were collected and counted at hCG24 hrs. Esr2-EdnraKO mice ovulated significantly fewer oocytes than WT controls, and had a greater range of oocytes ovulated. Error bars represent the SEM; diamonds indicate each data point. (**B**) Histology of representative ovaries at hCG24 hrs from each genotype, 4X. (**C**) WT and Esr2-EdnraKO female mice were bred with control proven males, one male and one female of each genotype per cage for 10 days. The percentage of females that gave births and the number of pups per litter quantified. There was no difference between groups. (**D**) Histology of representative ovaries after breeding study, 4x. Multiple corpora lutea were present in each group.

In mammals, endothelins exert their actions through two receptors, EDNRA and EDNRB. EDNRA has been previously implicated in EDN2 signaling and impaired ovulation via ovarian contraction^5, 6, 11, 56^. To first examine the hypothesis that ovulation is impaired without EDNRA signaling, Esr2-EdnraKO mice which lack *Ednra* in the granulosa cells were generated by crossing Esr2-iCre mice with floxed *Ednra* mice. Immature female mice were superovulated and oocytes were collected at hCG24 hours. Esr2-EdnraKO mice showed reduced ovulatory ability (10.2 ± 1.9 vs 21.7 ± 2.0 oocytes/ovary, p = 0.002) (Fig. 5A). Histologically, ovaries appeared similar to controls and possessed multiple corpora lutea as well as antral follicles (Fig. 5B). Esr2-EdnraKO mice also underwent a breeding study where one female and one WT female were paired together with the same male that had successfully sired previous litters (n = 4). There was no difference in fertility between WT and Esr2-EdnraKO mice, and there was also no significant difference in the number of pups per litter (5.0 ± 0.58 vs 4.7 ± 1.20, Fig. 5C). There was similarly no histological difference between ovaries following the fertility assay, and multiple CL were preset in each genotype (Fig. 5D). These data suggest that there is some EDNRA involvement in EDN2 signaling to granulosa cells, but the ovaries appear histologically different after superovulation from Esr2-Edn2KO ovaries. Although there was no difference in this fertility study from WT mice, the fecundity of Esr2-EdnraKO mice is similar to that of Esr2-Edn2KO mice (Fig. 3C). Therefore, *Ednra* expression in granulosa cells is involved in EDN2 signaling during ovulation, but is likely not the only important source of signaling.

### EDN2 induces contraction in mouse ovaries and uteri

**Figure 6:**
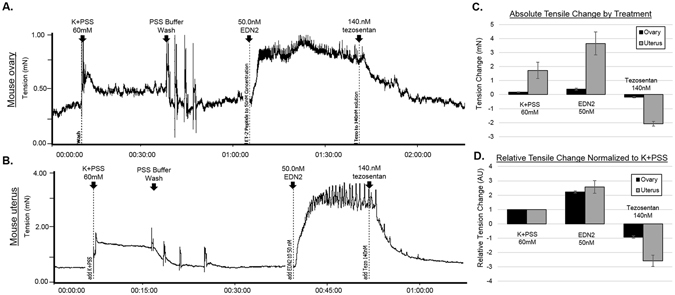
EDN2 induces contraction in mouse ovaries and uteri in an Endothelin receptor-dependent manner. WT mice were superovulated and ovaries (n = 8) were collected at hCG12–16 hours and were placed in a myograph machine for tension analysis. (**A**) A representative control ovary response to a 60 mM K + PSS solution (for normalization), a 50 nM EDN2 solution, and a 140 nM tezosentan solution to block Endothelin receptors. This treatment schema was similarly used to measure responses in (**B**) WT uteri (n = 5). Vertical axis: tension in millinewtons where 9.81 mN = 1.00 gram-force. Horizontal axis: Time (Hours:Minutes:Seconds). (**C**) Comparison of absolute tension changes between tissues in response to Endothelin receptor agonization and subsequent antagonization. (**D**) Ovarian and uterine contraction normalized to the response to K + PSS to compare relative contractile responses. Error bars represent the SEM.

To further explore the mechanism behind EDN2-mediated oocyte release, isometric contractions were performed on WT ovaries at hCG12–16 hours using a myograph tensile analysis. This is similar to previous studies performed on rat ovaries^5^ and confirms EDN2-induced contraction in mouse ovaries as well. First, the response of WT ovaries to different concentrations of EDN2 was demonstrated (Figure S3). Following mounting onto two pins, ovaries were normalized to 60 mM potassium in physiological saline solution (K + PSS) and then exposed to increasing concentrations of EDN2 in PSS, from 50 pM to 50 nM (n = 4). Contraction increased linearly in response to increasing doses of EDN2. At 50 nM, contraction response reached a near maximum with little increase in tension from previous concentration (Figure S3). Importantly, the 50 nM concentration was subsequently used to treat ovaries and uteri of WT mice to determine the contractile response (Fig. 6). This concentration agreed with previously employed values by Ko *et al*.^5^ in rats and allows for simple comparison. The average increase in tension from baseline in response to 50 nM EDN2 for a mouse ovary was 0.39 mN, or about 2.2 times the average response to K + PSS. Mouse uteri, which have significantly more smooth muscle and which also functioned as an internal control, demonstrated a greater tensile response to EDN2, 3.6 mN or about 2.6 times the average response to K + PSS. To confirm that this contraction was specific to endothelin receptors, the dual endothelin receptor antagonist tezosentan was added at a concentration of 140 nM after EDN2 and without washing away the EDN2 ligand solution. This concentration of tezosentan was previously shown to completely prevent EDN2-induced contraction when administered prior to EDN2 (data not shown). The average reduction in ovarian tension after tezosentan was 0.18 mN and −0.9 times the response to K + PSS; similarly, the response to tezosentan in uteri was 2.1 mN and −2.6 times the response to K + PSS (Fig. 6). Thus, EDN2 induces a consistent and strong contraction in both mouse ovaries and uteri that is greater in magnitude than treatment with a 60 mM potassium solution and which may be sufficient to induce oocyte expulsion during ovulation.

## Discussion

A surgical mouse model and multiple conditional knockout mouse models were used to demonstrate that *Edn2* is necessary for normal ovulation, and that this occurs through a contractile mechanism at the level of the ovary. Conditional deletion of *Edn2* in the ovary results in decreased ovulation, and consequently fewer pups per litter, fewer corpora lutea, and increased antral follicles with entrapped oocytes present within the ovary. To our best knowledge, this is the first study utilizing conditional *Edn2* loss within the murine ovary and the resulting effects on fertility and fecundity. These model animals are available for future utilization to investigate the underlying mechanisms of EDN2 and its induction as well.

In Edn2KO mice, systemic loss of *Edn2* causes early juvenile death likely through starvation, though ovarian development is only slightly affected by PND6. There was a trend towards a lower percentage of primordial follicles in Edn2KO ovaries, indicating that germ cell maturation into primordial follicles may be slighted retarded, yet generally proceeds similar to WT animals. Thus, the extraovarian effects of EDN2 are negligible for ovarian development in Edn2KO animals. Following surgical transplantation, ovarian function remained intact until the point of ovulation, though ovulation beneath the kidney capsule was not observed. Lack of blood flow to the ovary (ischemia) is the main complication following surgery that prevents ovarian survival^57–60^. Reformation of vessels takes more than 48 hours after ovarian grafting^61, 62^, but corpora lutea are present within 7 days in non-stimulated ovaries^63^. After transplantation, whole-ovarian loss of EDN2 does not prevent histologically identifiable CL formation in healthy adult mice, despite impaired follicle. The Edn2KO mice generated were similar to previous reports on global *Edn2* loss by Chang *et al*.^33^, though Edn2KO mice used in this study were generated by ablating the second exon which encodes the entire biologically active peptide sequence, which may account for the slightly shorter lifespan reported herein. Edn2KO ovaries transplanted to the kidney (KOT) had decreased ovulatory ability and had more trapped oocytes present than transplanted WT ovaries. These ovaries also had fewer CL than intact WT ovaries. The number of CL may be considered a marker for the number of potential ovulation, though it must be noted that the recipient mice were old enough to enter a natural estrus and present CL may be resultant from ovulation prior to superovulation induction as mouse CL persist for multiple estrous cycles^64^. Decreased *Edn2* expression in wtKOT mice was unexpected and may be a component of decreased vasculature present in the transplanted ovaries relative to intact age-matched mice. Of key note from this surgical experiment set is the increase in CL with trapped oocytes between WT KOT and Edn2KO KOT ovaries. Similar significant differences between these groups would likely have appeared with increased n-values, although this would require significant loss of life through surgery and non-viable Edn2KO mouse generation.

Granulosa cell-specific loss of *Edn2* at various times during folliculogenesis caused smaller litter size (subfecundity) and fewer oocytes ovulated (“subfertility”), although all mice retained the ability to give birth to healthy pups and to form morphologically normal corpora lutea. The range of severity varied with timing of Cre expression, which was likely because of the time required for Cre recombinase to remove *Edn2*. A new Esr2-iCre mouse successfully ablated the majority of ovarian *Edn2* expression and protein production during ovulation. Specific anti-EDN2 antibodies were not available and potential differences in locality of EDN2 could not be defined; EDN2 is a diffusible and very short peptide (21 a.a), and is likely difficult to be specifically detected by IHC. The Esr2-Edn2KO mice had few changes in gene expression at hCG12 hours, though ovarian EDN2 protein content was sharply reduced. Instead, the cause of subfertility occurred through the normal contractile and vasoconstrictive properties of EDN2. Mice subsequently had more antral follicles and fewer corpora lutea. These data support that EDN2 acts as a tensile trigger for ovulation by causing contraction in either the theca externa layer or the associated vascular smooth muscle cells. The loss of the oocyte from the AF or blood vessel rupture are likely similarly important for induction of luteinization. Speculatively, EDN2 may also influence tension in the oviduct or uterus to increase the frequency or intensity of their continual contractions for fluid or germ cell transport. Future studies may focus on the physiological role of EDN2 by supplementing endothelins to conditional knockout mice during ovulation compared to other contractile agents that act through unchanged receptor pathways. Importantly, this study also compares various granulosa-cell specific Cre mouse lines; when removing gene expression in mature ovaries, Esr2-iCre had superior efficacy to Pgr-Cre and Cyp19-iCre in reduction of *Edn2* expression. Though Cyp19-iCre seems to trend towards a decrease in the number of oocytes ovulated, the significant decrease in pups per litter indicates either that loss of *Edn2* at the time of aromatase expression is sufficient to prevent normal follicle rupture, or that Cyp19-iCre expression has incomplete removal of *Edn2* throughout all granulosa cells; the latter is supported through immunohistochemical staining (unpublished). Another point of this conditional knockout experiment to consider is the extraovarian expression of *Edn2*, specifically in the oviduct and uterus. Though it would not impact ovulation, phenotypic decreased fecundity may be partially dependent on changes in EDN2 in these organs.

Generation of Esr2-EdnraKO mice supports minor involvement of EDNRA in EDN2-mediated follicle rupture in granulosa cells. These mice ovulated fewer oocytes and gave birth to relatively small litters, though control mice also gave birth to fewer pups than would be expected for C57B/6 mice. Esr2-EdnraKO mice did not have as severe deficits as Esr2-Edn2KO mice. A less severe phenotype is also supported by ovarian histology, which indicates that many CL were present and relative few antral follicles or antral follicles with trapped oocytes were present. As Esr2-iCre removed *Ednra* in the granulosa cells^40^, EDN2 may have a minor function in granulosa cells that is not critical for ovulation. This granulosa cell effect may impact ovulation partially or indirectly, but it is more likely EDN2 signaling through smooth muscle receptors that has a severe and important impact on ovulation.

This study further presents data on mouse ovarian contraction relative to EDN2 treatment which replicates previous rat data. Work by Ko *et al*.^5^ demonstrate that rat ovaries contract in response to EDN2 at a 50 nM dose and that this tension can be modified by treatment with tezosentan at 605 nM (10 mg/mL) as well, when compared to a K + control. The presented data show that EDN2 at 50 nM produces a similar relative response in mouse ovaries and that tezosentan reduces this contraction, though at approximately 15% the dose of tezosentan previously reported. The dose of EDN2 used for contraction in these experiments was equivalent to that used in previous rat studies.

In conclusion, the perspective that EDN2 is crucial for ovarian contraction and subsequent ovulation adds to a more comprehensive knowledge of the ovulatory process, and may contribute in the future to contraceptive or pro-fertility treatments. EDN2, or its receptors, may be inhibited in a potential novel non-hormonal contraceptive, or ovarian vasoconstriction may be stimulated to help women undergoing artificially induced ovulation treatment.

## Methods

See SI Materials and Methods for detailed methods. This study was carried out in accordance with the recommendations in the Guide for the Care and Use of Laboratory Animals of the National Institutes of Health. Animal protocol was approved by the University of Illinois Animal Care and Use Committee (Protocols: 11184, 12090, 13032, 14222, and 14247), and all efforts were made to minimize animal suffering. Results are expressed as mean ± SEM.

### Animals used

Mice were generated by crossing Edn2flox/flox mice purchased from Jackson Laboratory^65^ with Pgr-Cre mice^34^, Cyp19-iCre mice^35^, and knock-in Esr2-iCre mice^40^, or with Zp3-Cre mice^32^ to generate global *Edn2* knockout mice (Edn2KO). Ednraflox/flox mice were bred with Esr2-iCre mice^66^ to remove *Ednra* in the granulosa cells.

### Kidney-Ovarian Transplantation (KOT)

Ovaries were grafted under the kidney capsule following methods developed by Jackson Laboratories. The recipient female mice, at the age of 30 days, were anesthetized by isoflurane and an incision was made between the last rib and the iliac crest. Ovaries were removed after clamping the ovarian blood vessels (ovariectomy). The kidney was exposed through the same incision site and the post-natal day six (PND6) donor ovary was pushed through the incision and pushed to the opposite pole of the kidney. The incision was sutured closed and repeated on the opposite side. Mice were allowed to recover for 19 days post-surgery.

### Fertility assay, Superovulation, RT-PCR, and Histology

For fertility assay, each set of females was paired for 10 days with a proven WT male breeder, then separated and monitored daily. Litter size describes the number of pups present at birth; age refers to the start of mating period. Superovulation was performed by single injection of 5IU PMSG and 5IU hCG at 48 hour intervals to 25 day old mice (ovarian age 25 days in ovarian transplant mice). Mice were sacrificed 12 or 24 hrs after hCG injection. At 12 hrs after, RNA was extracted and purified. Complementary DNA was generated and qRT-PCR was done for *Edn1* and *Edn2*, with *Rpl19* as an internal control. Data were analyzed using the delta delta Ct^67^ method. For histology, ovaries from hCG24 hours were fixed, embedded in paraffin, sectioned at 5 μm, and stained with hematoxylin and eosin for follicle counting. AF were histologically defined as spaces containing an oocyte with continuous fluid 100 um in diameter; CL were defined as cellular and/or partially eosinophilic fluid-filled spaces lined by polygonal eosinophilic cells containing lipid droplets.

### Endothelin Protein Quantification

Soluble endothelins were extracted from hCG12 hrs ovaries with 1 M HCl in 100% ethanol. After ethanol evaporation, a commercial ELISA kit (583151 Cayman Chemical) was run according to the manufacturer’s instructions. Plates were read at 405 nm and analyzed with a quadratic standard curve.

### Serum Steroid Measurement

Samples were prepared by removing the serum supernatant. Ten μL of mouse serum was mixed with 50 μL methanol and 1 μL of 2 μg/mL D9-progesterone. The supernatant was subjected to LC/MS/MS injection at the Metabolomics Center at the University of Illinois at Urbana-Champaign. Double charcoal-stripped steroid free mouse serum was used as a standard. For detection, samples were analyzed with the 5500 QTRAP LC/MS/MS system (AB Sciex, Foster City, CA) in Metabolomics Lab of Roy J. Carver Biotechnology Center.

### Ovary Mounting and Isometric Tension Measurement

Whole ovaries used for tension analysis were placed into a physiological saline solution (PSS) at 37 °C in a wire myograph system (Tissue Bath System 620 M, Danish Myo Technology, DMT-USA Inc., Ann Arbor, MI). Each ovary was mounted with 4 mm pins on each myograph arm. Ovarian tensile measurements were recorded via LabChart software with a constant tension of 1 mN. A ‘wake-up’ protocol was used for equilibration: K + PSS was applied to the stretched ovary, and was removed 3 min later. The ovary was then washed 4 times over 5 min with PSS, and allowed to sit for 5 more min. This procedure was repeated twice. The contractile experiment began at time zero. K + PSS solution was applied a 3^rd^ time for a duration of 5 min to serve as a reference point. K + PSS was then removed with four PSS washes. Next, human endothelin-2 purified peptide (American Peptide Co, Sunnyvale, CA) was added to the PSS solution at a 50 nM concentration. After 20 min. the dual-endothelin receptor antagonist drug tezosentan (tezo) was added to a concentration of 140 nM. Fifty nM of EDN2 was the lowest dose that consistently produced the strongest contraction (Figure S3). Similar methods were used for uterine tissue mounted longitudinally between two pins.

### Statistical Analysis

Data analyses were performed using statistical software (SPSS 22.0, Chicago, IL). Continuous data were tested for normal distribution by a Shapiro-Wilk test. Following homogeneity of variance confirmation, all normally distributed continuous data were analyzed with parametric tests and a Bonferroni *post hoc* test. All non-normally distributed continuous data were transformed by log function to a normal distribution if possible or analyzed by non-parametric tests (Mann Whitney U, Kruskal Wallis ANOVA). Ordinal data were similarly analyzed. Data are graphically presented as the mean and standard error of the mean unless otherwise indicated. For all analyses the alpha value was set to 0.05.

## Electronic supplementary material

Supplemental Information
