# Supplementary material for: Granulosa cell endothelin-2 expression is fundamental for ovulatory follicle rupture
Source: Sci Rep. 2017 Apr 11;7:817. doi: 10.1038/s41598-017-00943-w (PMC5429765; doi:10.1038/s41598-017-00943-w)
Supplement: Supplementary file 1 — Supplemental Information [file 41598_2017_943_MOESM1_ESM.doc]

**Granulosa cell endothelin-2 expression is fundamental for ovulatory follicle rupture**

**Supplemental Information**

Joseph A. Cacioppo^1^, Po-Ching Patrick Lin^1^, Patrick R. Hannon^1,2^, Daniel R. McDougle^1^, Arnon Gal^1,3^, and CheMyong Ko^1^

^1^Department of Comparative Biosciences, College of Veterinary Medicine, University of Illinois at Urbana-Champaign, Urbana IL 61802, USA;

^2^Department of Obstetrics & Gynecology, University of Kentucky, Lexington, KY, 40536, USA;

^3^Department of Small Animal Internal Medicine, Institute of Veterinary, Animal and Biomedical Sciences, Massey University, Palmerston North, New Zealand 4442

Corresponding author:

CheMyong Jay Ko, PhD

Department of Comparative Biosciences

College of Veterinary Medicine

University of Illinois at Urbana-Champaign

2001 S. Lincoln Ave.

Urbana, IL 61802

Phone: 217-333-9362

Fax: 217-244-1652

[JayKo@illinois.edu](mailto:JayKo@illinois.edu)

**Supplemental Figures**


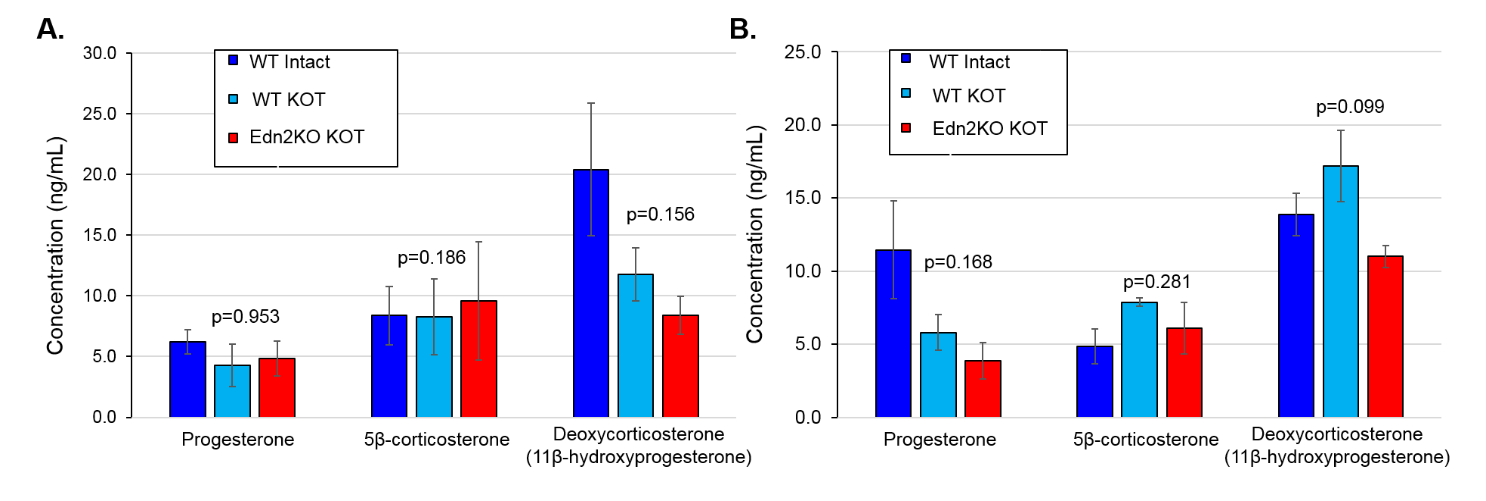


**Fig. S1. Serum hormones are not different between ovarian graft mice and intact mice.** (*A*) Serum was extracted from mice at hCG12 and (*B*) hCG24 hours. Serum hormone concentrations (ng/mL) were not different between intact WT mice, ovariectomized (OVX) WT mice with one WT ovary grafted to each kidney, or WT mice with one Edn2KO ovary grafted to each kidney. P-values represent ANOVA score by hormone. Error bars represent the SEM.


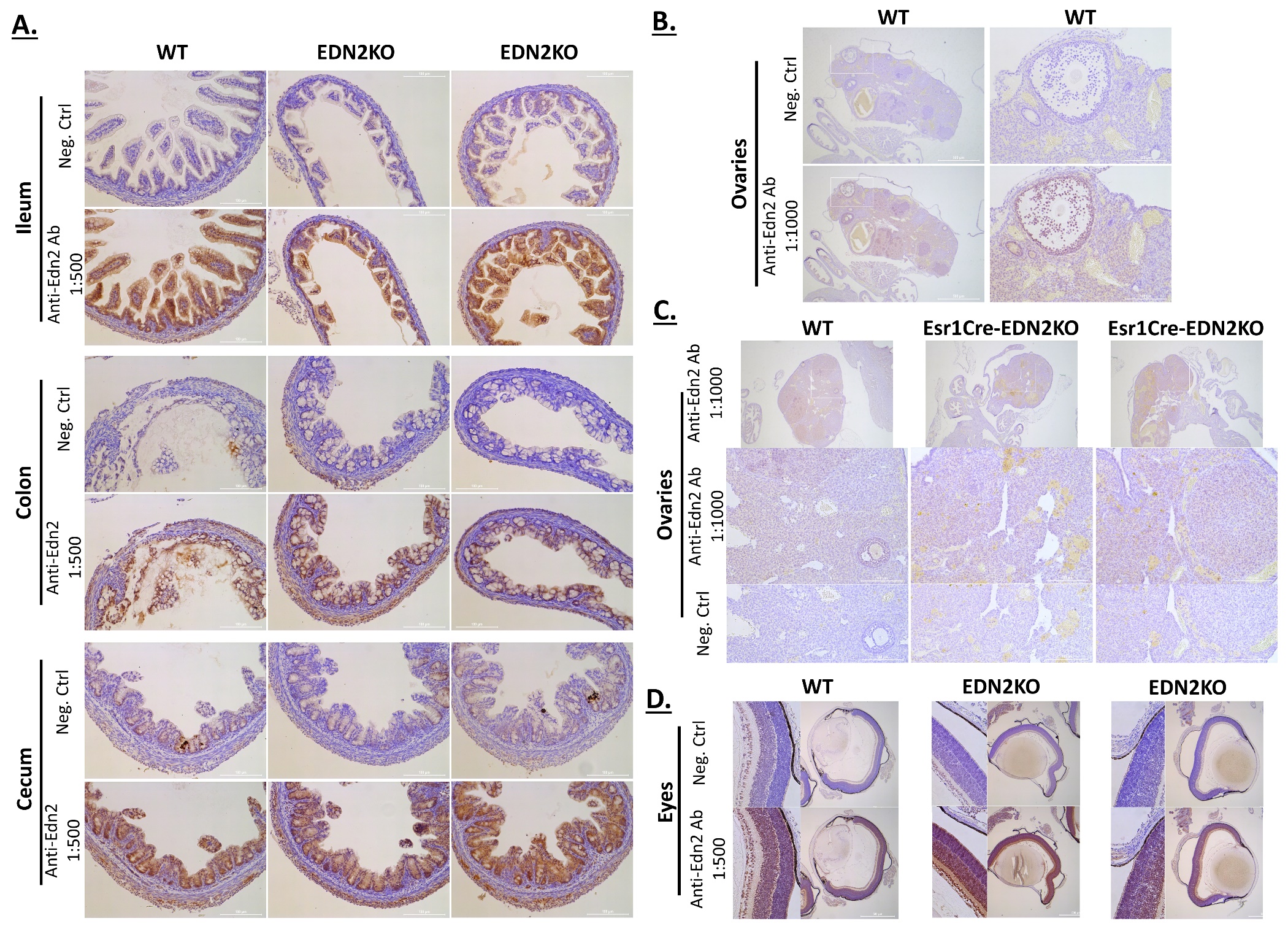


**Fig. S2.**

**Fig. S2. Anti-EDN2 antibody staining is nonspecific.** WT, global EDN2KO, and Esr1Cre-EDN2KO mouse tissues were stained with rabbit anti-EDN2 antibody (Abcam 197763). (*A*) Both WT and global EDN2KO gastrointestinal tissues stain positively with anti-EDN2. Particularly prominent staining is present in the mucous cells of the villi of the ileum and the muscularis layer of the colon. (*B*) WT ovarian IHC is nonspecific for anti-EDN2 with staining present outside the granulosa cells of mature follicles. Ovarian stromal cell EDN2 staining is not supported by previous studies. (*C*) Staining with anti-EDN2 is similar between WT and granulosa cell-specific EDN2KO tissues. (*D*) Retinal staining with anti-EDN2 antibody is similar between WT and global EDN2KO tissues. Staining is present within the nuclear and plexiform layers of both retina of each genotype. All images shown were taken at 40X or 200X and scale bars indicate 500 or 100 µm, respectively.


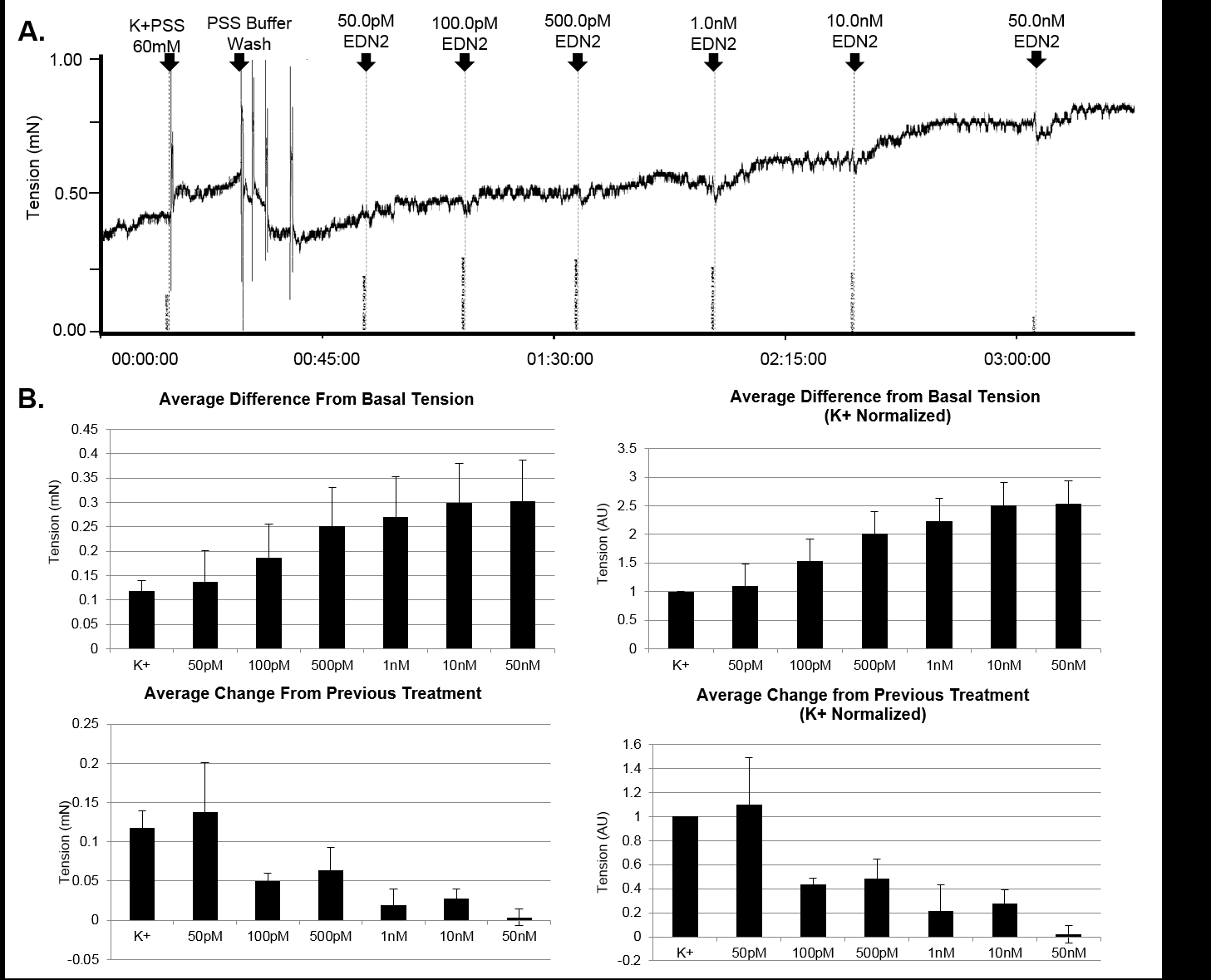


**Fig. S3. WT mouse ovaries contract maximally in response to 50nM EDN2.** WT mice (n=4) were superovulated at 28 days old and ovaries were collected at hCG12-16 hours. Ovaries were placed in a myograph machine for tension analysis. (*A*) A representative ovary response to a 60mM K+PSS solution (for normalization) and increasing concentrations of EDN2 solution. This ovary demonstrates a contraction in response to EDN2 that increases with concentration to 50nM. Vertical axis: tension in millinewtons where 9.81mN = 1.00gram-force. Horizontal axis: Time (Hours:Minutes:Seconds). (*B*) Comparison of tension between concentrations with absolute (left) and relative (right) responses to endothelin receptor agonization by EDN2. Ovarian contraction was normalized to the response to K+PSS to compare relative contractile responses. The lowest increase in tension occurs at 50.0nM EDN2, indicating near saturation of receptors. Error bars represent the SEM. This treatment schema was similarly used to measure responses from WT uteri, where EDN2 at 50.0 induces a strong contraction near the receptor saturation point, approximately 3.0 times as strong as the contraction from a 60mM K+ solution.

**SI Materials and Methods**

*Ethics Statement*

This study was carried out in strict accordance with the recommendations in the Guide for the Care and Use of Laboratory Animals of the National Institutes of Health. Animal protocol was approved by the University of Illinois Animal Care and Use Committee (Protocols: 11184, 12090, 13032, 14222, and 14247), and all efforts were made to minimize animal suffering.

*Animals used*

Animals were maintained under a 12 hour light-dark cycle and given a continuous supply of Harlan rodent chow (Indianapolis, IN) and water. Mice were generated by crossing Edn2flox/flox mice purchased from Jackson Laboratory[^1^](#_ENREF_1) (Bar Harbor, Maine) with Pgr-Cre mice[^2^](#_ENREF_2), Cyp19-iCre mice[^3^](#_ENREF_3), and knock-in Esr2-iCre mice[^4^](#_ENREF_4) to remove *Edn2* specifically in the granulosa cells at various time points during folliculogenesis, or with Zp3-Cre mice[^5^](#_ENREF_5) to generate global *Edn2* knockout mice (Edn2KO). Edn2flox/flox mice were also crossed with novel Esr1-Cre mice (*in publication*) to remove *Edn2* from the granulosa cells of the ovary for immunohistochemistical anti-Edn2 antibody validation. Ednraflox/flox mice were bred with Esr2-iCre mice to remove *Ednra* in the granulosa cells. Genotyping was performed on ear biopsies using the HotShot method[^6^](#_ENREF_6). The following primer sets were used for genotyping:

| Edn2Flox_F | CAT AGA GCG GTG AGG CCA CAG | Flox: 170bp |
| --- | --- | --- |
| Edn2Flox_R | AAG TTG GCA CCC TTG GTG TTC | WT: 130bp |
| Edn2KO_R | CTG TTC AGC TGG CAG AGT GAA GC | KO: 400bp |
| Ednra_F | CCTCAGGAAGGAAGTAGCAAGATTA | Flox: 650bp |
| Ednra_R | ACACAACCATGGTGTCGA | WT: 610bp |
| Ednra_F2 | GAGAACCTACAACTGGGGACACAAACAC | KO: 1200bp |
| Esr2-iCre_F | CAG GTG CTG TTG GAT GGT CTT C | Esr2-iCre: 401bp |
| Esr2-iCre_R1 | CTT AGT TAC TCC GGC AGC TTG AAC | WT: 181bp |
| Esr2-iCre_R2 | AGG GGA AGT AAG GCT TGA TGG TGA |  |
| iCre_F | TCT GAT GAA GTC AGG AAG AAC C | iCre: 500bp |
| iCre_R | GAG ATG TCC TTC ACT CTG ATT C | WT: no band |
| Pgr-Cre_F1 | ATG TTT AGC TGG CCC AAA TG | PR-Cre: 594bp |
| Pgr-Cre_F2 | CCC AAA GAG ACA CCA GGA AG | WT: 283bp |
| Pgr-Cre_R | TAT ACC GAT CTC CCT GGA CG |  |
| Zp3-Cre_F | GGA CAT GTT CAG GGA TCG CCA GGC G | Zp3-Cre: 250bp |
| Zp3-Cre_R | GTG AAA CAG CAT TGC TGT CAC TT | WT: no band |

*Kidney-Ovarian Transplantation (KOT)*

Ovaries were grafted under the kidney capsule following methods developed by Jackson Laboratories. Ovaries from post-natal day six (PND6) mice were removed following CO_2_ euthanasia. The recipient female mice were anesthetized by continuous nasal inhalation of isoflurane (2-2.5%) with 1 liter per minute (LPM) of oxygen flow. After appropriate depth of anesthesia was reached, fur was removed from an area centered over the proposed incision sites, 15 mm wide by 30 mm long. The size of the area clipped was proportional to the size of the mouse. Skin was disinfected with surgical iodine and 70% ethanol using sterile applicators. Eye gel/ointment was applied to both eyes to prevent dehydration. The mouse was placed on a heat pad (37C) to prevent hypothermia during surgery. The donor mouse was placed in ventral-lateral recumbency. A 4-7mm skin incision was made parallel and ventral to the spine midway between the last rib and the iliac crest. An incision was made in the underlying abdominal wall. Forceps were used to spread open the incision to look inside the abdominal cavity and locate the ovary. Forceps were used to grasp and exteriorize the ovarian fat pad. The fat pad was positioned on a 2x2 Versalon sterile sponge so that the ovary was facing the surgeon and the oviduct was ventral. A Moria forceps was placed under the ovary (medial aspect) to clamp the ovarian blood vessels. A second pair of Moria forceps was placed immediately proximal to the first pair and used to shear the ovary off of the ovarian blood vessels. Before releasing the first forceps, one drop of epinephrine was added to the vascular stump for one minute. The collected ovary was discarded and the fat pad was returned to the body. Next the kidney was exposed through the same incision site and a 2mm incision was made through the kidney capsule on one pole of the kidney. The donor ovary was pushed through the incision and pushed to the opposite pole of the kidney. The kidney was returned to the abdominal cavity. The incision in the abdominal wall was closed with 5-0 or 6-0 absorbable suture with a swaged on needle. The skin incision was closed with the same suture in a simple interrupted pattern or with wound clips. The same procedure was repeated on the opposite side of the mouse to produce a WT ovariectomized mouse (OVX) with one Edn2KO ovary under each kidney capsule. Control mice were also produced with WT ovaries inserted under the kidney capsule. Mice were allowed to recover and skin suture or wound clips were removed 7 days later. Post-surgical mice were transferred to a clean, warm cage. Mice were placed on a paper towel in ventral or lateral recumbency with head slightly extended. A paper towel was placed under the rodent to minimize bedding adhering to the incision site. Each mouse was given a subcutaneous injection of warm sterile saline (1-2 cc/25g body weight) to avoid dehydration. For pain control, carprofen 5mg/kg was injected subcutaneously. Temperature in the recovery area was controlled to prevent hypothermia during recovery. Supplemental heat was provided by placing the recovery cage on a slide warmer. The cage was warmed to 80-86°F (26.7-30°C) and the animal was provided a means to move away from the heat source once awake. The anesthetized mouse was observed at least every 10 minutes until it was able to move about the cage on its own to ensure that it did not crawl into a corner and obstruct its nose. Frequent stimulation such as touching and moving the mouse was performed. The followings were interpreted as signs of pain or distress: constant facial expression of discomfort (orbital tightening, nose bulge, cheek bulge, and "pulled back" ear position), facial wiping with the forelimb, licking, biting, and scratching the surgical wound, and additional carprofen was administered every 12 hours.

*Vaginal Cytology*

Vaginal cytology was performed according to guidelines set by Gal et al.[^7^](#_ENREF_7). Briefly, mice were smeared daily at approximately 1100 hours by application of 100uL of PBS into the distal aspect of the vagina followed by aspiration of fluids. Samples were placed into a clear 96-well plate and read using an inverted microscope (Olympus CKX41, Center Valley, PA). Proestrus (P) was characterized by predominantly round to polygonal cells that occasionally have a discernable nucleus with several leukocytes; Estrus (E) was characterized by many anucleate polygonal cells and a lack of leukocytes; Metestrus (M) was characterized by a combination of an equivalent number of leukocytes and anucleate polygonal cells; and Diestrus (D) was characterized by a marked dominance of small, round leukocytes.

*Fertility assay*

This assay was conducted to determine if mice were capable of giving birth to a normal number of litters with a normal number of pups and gender distribution. Adult female mice were randomly allocated into groups of two or three females of each treatment. Each set of females was paired for 10 days with a proven WT male breeder, aged 3 months – 7 months. Females were then removed from the male and separated into individual cages for 21 days and monitored daily. The percentage of females that gave birth from each treatment group, the average number of pups per litter, and the gender distribution of the pups was recorded. Litter sizes describe the number of pups present at birth. Females repeated the breeding assay two to four times. Average age describes the age at the start of mating period.

*Superovulation***,** *RT-PCR, and Histology*

Superovulation was performed by single intraperitoneal (i.p.) injection of gonadotropins (5IU pregnant mare serum gonadotropin, PMSG, and 5IU human chorionic gonadotropin, hCG) at 48 hour intervals to 25 day old mice. Mice were sacrificed 12 or 24 hrs after hCG injection. At 12 hrs after, ovaries were frozen, RNA was extracted using Trizol® solution (Ambion, Carlsbad, CA), and then purified with a Qiagen RNEasy Kit (Valencia, CA). RNA was analyzed by a Nanodrop machine for quantity and quality. Complementary DNA was then generated by M-MLV Reverse Transcriptase using random primers. Template RNA quantities were normalized to 1.0μg/μL prior to reverse transcrpition. 1.0μL of resulting cDNA from each ovary was used as template for semi-quantitative RT-PCR with 15μL Taq Platinum (Invitrogen) and 0.3μL of each primer per PCR reaction; reactions were cycled 30x for 94C for 1:00 min, 53C for 1:00, and 72C for 1:30. Amplified DNA (3.0 μL) was visualized on a 2.0% agarose gel and was quantified using ImageJ freeware (NIH, Bethesda, MD) to measure peak pixel grayscale levels within the defined areas of the bands. The ribosomal 60S subunit L19 (*Rpl19* gene; L19) was used as an internal control with (5'-CCTGAAGGTCAAAGGGAATGTG-3' and 5'-GTCTGCCTTCAGCTTGTGGAT-3'). Expression of *Edn2* was analyzed with primers (5’-CTCCTGGCTTGACAAGGAATG-3’ and 5'-GCTGTCTGTCCCGCAGTGTT-3'). Expression of *Edn1* was analyzed with primers (5'-AGCCGAACTCAGCACCGGAGCT-3' and 5'-ATGATGTCCAGGTGGCAGAAGTAGAC-3'). For quantitative RT-PCR using the Taqman system (Life Technologies), primers Mm0432983_m1 were purchased for *Edn2* and primers Mm02601633_g1 for *Rpl19* (Life Technologies). Taqman reactions were run on an ABI 7500 machine and data were analyzed using the delta delta Ct[^8^](#_ENREF_8) method. After fixation in the 4% PFA, ovaries from hCG24 hours were embedded in paraffin and sectioned at 5 μm, mounted on charged glass slides, deparaffinized, and rehydrated. Tissues were then stained with hematoxylin (Harris) and eosin (Surgipath) for cellular visualization for use in follicle counting.

*Endothelin Protein Quantification*

Soluble endothelins were extracted following protocol of Chakravarthy *et al*.[^9^](#_ENREF_9) and measured following protocol by Choi *et al*.[^10^](#_ENREF_10). Briefly, individual whole ovaries from hCG12 hours were placed in individual 1.5mL tubes with 500uL 1M HCl in 100% ethanol, homogenized with a mortar and pestle, and incubated at 4C for 48 hours on an orbital shaker. Tubes were centrifuged at 2000xg for 30 minutes, then liquid was transferred to a clean tube. Ethanol was then removed by 1 hour incubation in an unheated vacuum centrifuge to dry protein. To measure endothelin quantity, a commercial ELISA kit (583151) was purchased from Cayman Chemical Company (Ann Arbor, Michigan). Individual ovarian endothelins were constituted with 500uL of EIA buffer and loaded onto the plate, and plates were run according to the manufacturer’s instructions. Plates were read at 405nm and analyzed with a quadratic standard curve.

*Immunohistochemistry*

Three previously published manuscripts reference two anti-EDN2 antibodies; one antibody for the earliest two manuscripts was privately produced through the Saida laboratory and is not available. The more recently published and commercially available rabbit anti-EDN2 antibody (Abcam 197763)[^13^](#_ENREF_13) was subsequently purchased with the intent of localizing EDN2 peptide. The specificity of this antibody was tested using tissue sections freshly prepared from WT, global Edn2KO, and novel Esr1-Edn2KO mice (*in publication*: granulosa cells lose EDN2 expression); no fresh tissue was available from Esr2-Edn2KO mice at the time of study. Murine ovary, oviduct, uterus, eye, ileum, cecum, and colon tissue were collected from freshly-killed animals, rinsed in PBS, and were fixed for 48 hours in a 4% paraformaldehyde solution. Tissues were then embedded in paraffin and sectioned at 5 μm, mounted on charged glass slides, deparaffinized, and rehydrated. Antigen retrieval was done with Sodium Citrate Buffer pH 6.0 for 10 minutes at 100°C. Endogenous peroxidase activity was quenched by 3% H2O2 for 30 minutes. Sections were blocked with 2% goat serum (PK-6101) for 60 minutes. Trials where avidin/biotin blocking (4 drops/1ml (SP-2001)) were also attempted. Sections were then treated with the primary antibody (Rabbit Anti-Edn2 Ab: abcam197763) or negative control PBS solution of equal volume. Primary antibody concentrations were varied from 1:500 to 1:1000 to maximize specificity. Primary antibody staining was done at 4°C overnight (13 hours). Sections were then washed with PBST and the secondary antibody (Goat anti-rabbit IgG from PK-6101) was treated for 10 minutes. Visualization was performed with fresh Avidin Biotin complex solution for 20 minutes at room temperature followed by DAB for 2 minutes prior to dilute hematoxylin counterstaining.

*Serum Steroid Measurement*

Progesterone was considered the principle hormone measured and all others were secondary. Samples were prepared by spinning down whole blood after clotting and removing the serum supernatant. 10uL of mouse serum was mixed with 50μL methanol and 1μL of 2 ug/mL D9-progesterone, followed by the rigorous centrifugation. The supernatant was subjected to LC/MS/MS injection at the Metabolomics Center at the University of Illinois at Urbana-Champaign by Dr. Lucas Li. The standard solutions for the calibration curve used double charcoal-stripped steroid free mouse serum provided by Dr. Kee Jun Kim and followed the same extraction protocol as the serum samples. For detection, samples were analyzed with the 5500 QTRAP LC/MS/MS system (AB Sciex, Foster City, CA) in Metabolomics Lab of Roy J. Carver Biotechnology Center, University of Illinois at Urbana-Champaign.  The 1200 series HPLC system (Agilent Technologies, Santa Clara, CA) includes a degasser, an autosampler, and a binary pump. The LC separation was performed on an Agilent Zorbax SB-Aq column (4.6 x 50mm, 3.5μm.) with mobile phase A (0.1% formic acid in water) and mobile phase B (0.1% formic acid in acetonitrile). The flow rate was 0.3 mL/min. The linear gradient was: 0-2 min, 60%A; 6-12 min, 0%A; 12.5-17 min, 60%A. The autosampler was set at 5°C. The injection volume was 5 μL. Mass spectra were acquired under positive electrospray ionization (ESI) with the ion spray voltage of 5500 V. The source temperature was 550°C. The curtain gas, ion source gas 1, and ion source gas 2 were 38, 50, and 65, respectively. Multiple reaction monitoring (MRM) was used to measure progesterone (m/z 315.1-->m/z 97.1) with D9-progesterone used as internal standard (m/z 324.1 --> m/z 100.1).

For additional steroid profiling, samples were prepared by using 20 uL mouse serum sample mixed with 40μL methanol and 1μL 2μg/mL D9-progesterone, followed by the rigorous centrifugation. The supernatant was subject to LC/MS/MS injection. The standards solutions for the calibration curve again used double charcoal-stripped steroid free mouse serum, and followed the same extraction protocol as the serum samples. Samples were again analyzed with the 5500 QTRAP LC/MS/MS system (AB Sciex, Foster City, CA) in Metabolomics Lab of Roy J. Carver Biotechnology Center, University of Illinois at Urbana-Champaign.  The LC separation was performed on a Phenomenex C6 Phenyl column (2.0 x 100mm, 3μm.) with mobile phase A (0.1% formic acid in water) and mobile phase B (0.1% formic acid in acetonitrile). The flow rate was 0.25 mL/min. The linear gradient was as follows: 0-1 min, 80%A; 10 min, 65%A; 15 min, 50%A; 20 min, 40%A; 25 min, 30%A; 30 min, 20%A; 30.5-38 min, 80%A. The autosampler was set at 5°C. The injection volume was 5 μL. Mass spectra were acquired under positive electrospray ionization (ESI) with the ion spray voltage of 5500 V. The source temperature was 500 °C. The curtain gas, ion source gas 1, and ion source gas 2 were 36 psi, 50 psi, and 65 psi, respectively. Multiple reaction monitoring (MRM) was used to measure steroids with the following transitions (Q1-->Q3) with D9-progesterone as internal standard (m/z 324.1 --> m/z 100.1). Progesterone Q1 (m/z): 315.1, Q3 (m/z): 97.0; 5β-corticosterone Q1 (m/z): 349.2, Q3 (m/z): 313.2; Deoxycorticosterone/11β-hydroxyprogesterone Q1 (m/z): 331.2, Q3 (m/z): 97.1; Pregnenolone Q1 (m/z): 317.1, Q3 (m/z): 299.0. Although 24 different serum hormones were originally tested, including estrogen and testosterone, only those three hormones displayed were present at detectable levels.

*Ovary Mounting*

Ovaries used for tension analysis were placed into a physiological saline solution (PSS) at 37°C in a wire myograph system (Tissue Bath System 620M, Danish Myo Technology, DMT-USA Inc., Ann Arbor, MI). Whole ovaries were used after the removal of ovarian vessels, and pin mounting was chosen to minimize tissue damage and increase mounting speed. Each ovary was punctured on each end by one of the 4mm mounting pins on each arm of the myograph. The myograph was then turned on, and a mixture of 50% Oxygen, 45% Nitrogen, and 5% Carbon Dioxide was bubbled through the chamber. Two ovaries were processed at the same time for each set of experiments. Ovarian tensile measurements were recorded via LabChart software (ADInstruments, Colorado Springs, CO). After 20 min when the ovary in the test chamber had reached the set appropriate temperature, the PSS solution was removed and fresh heated PSS buffer was added. The system was then zeroed. Tension was next repeatedly applied to the half ovary over the following 20 min to maintain a constant tension of 1mN. Optimal tension-contraction response in ovaries occurred with ~1mN of passive tension applied per whole ovary. Lastly, a ‘wake-up’ protocol was used to finish the equilibration: K+PSS was applied to the stretched ovary, and was removed 3 min later. The ovary was then washed 4 times over 5 min with PSS, and allowed to sit for 5 more min. This procedure was repeated twice. The PSS was then replaced a final time and the contractile experiment began, time zero.

*Isometric Tension Measurement*

To determine the strength of ovarian contraction in response to endothelin-2 protein (EDN2) agonization, ovaries were first allowed to sit in fresh PSS at a physiological temperature and pH with supplied oxygen and CO_2_ for five minutes to generate a ‘baseline’ tensile measurement, the average tension in mN during this treatment. For ovaries that demonstrated cyclical contractions during this period, baseline measurements were averages of the trough tension for five troughs between peaks. Next, K+PSS solution was applied a 3rd time for a duration of 5 min. This served as a reference point for the relative contractility of that ovary to account for variation in mounting and ovary size. Any ovary that failed to demonstrate contraction in response to K+PSS was discarded. K+PSS was then removed with four PSS washes at 1 min intervals, and the ovary was allowed to equilibrate for 5 additional minutes. Next, human endothelin-2 purified peptide (American Peptide Co, Sunnyvale, CA) was added to the PSS solution of the chamber to generate a 50nM solution. After 20 min, without changing the buffer solution or removing the ligand, the dual-endothelin receptor antagonist drug tezosentan (tezo) was added to a concentration of 140nM and left for 20 minutes. These concentrations were calculated from titration experiments in which increasing amounts of EDN2 peptide were added to ovaries and the relative change in tension was recorded. 50nM of EDN2 was the lowest dose that consistently produced the strongest contraction (Figure S2). Tensile measurements represent the difference from resting measurement (the average of 5 minutes prior to the final K+PSS addition) to either average K+ contraction (the average of minutes 2:00-5:00 after the K+PSS addition at 0:00) or average EDN2 contraction (the average of the 5 minutes prior to tezosentan administration for EDN2-induced contraction). The average of the final 5 minutes of the experiment was subtracted from peak EDN2-induced tension to calculate the change after tezosentan addition. All measurements were made in mN, and were later converted to arbitrary units (AU) for relative comparison by dividing each by the contractile response to K+PSS for that ovary. Titrations of EDN2 or tezosentan to determine minimum concentrations for optimum effect were made as above, except varying doses of each were added in 20 minute intervals without washing away previous buffer solution to those ovaries. Similar methods were used for uterine tissue mounted longitudinally between two pins.

*Statistical Analysis*

Data analyses were performed using statistical software (SPSS, Inc., released 2013, PASW Statistics for Windows, Version 22.0, Chicago, IL). Continuous data were tested for normal distribution by a Shapiro-Wilk test. All normally distributed continuous data were analyzed with parametric tests (student’s t-test, ANOVA, or paired student’s t-test) and a *post hoc* test: the Bonferroni post hoc test was chosen owing to the small sample sizes available (although there was no variability in those data that were significantly different between Bonferroni or Tukey tests). All non-normally distributed continuous data were transformed by log function to a normal distribution if possible or analyzed by non-parametric tests (Mann Whitney U, Kruskal Wallis ANOVA). Ordinal data were similarly analyzed. Data are graphically presented as the mean and standard error of the mean unless otherwise indicated. For all analyses the alpha value was set to 0.05.

**SI Materials and Methods References**:

1 Rattner, A., Yu, H., Williams, J., Smallwood, P. M. & Nathans, J. Endothelin-2 signaling in the neural retina promotes the endothelial tip cell state and inhibits angiogenesis. *Proc Natl Acad Sci U S A* **110**, E3830-3839, doi:10.1073/pnas.1315509110 (2013).

2 Soyal, S. M. *et al.* Cre-mediated recombination in cell lineages that express the progesterone receptor. *Genesis* **41**, 58-66, doi:10.1002/gene.20098 (2005).

3 Fan, H. Y. *et al.* Selective expression of KrasG12D in granulosa cells of the mouse ovary causes defects in follicle development and ovulation. *Development* **135**, 2127-2137, doi:10.1242/dev.020560 (2008).

4 Cacioppo, J. A. *et al.* Generation of an estrogen receptor beta-iCre knock-in mouse. *Genesis* **54**, 38-52, doi:10.1002/dvg.22911 (2016).

5 de Vries, W. N. *et al.* Expression of Cre recombinase in mouse oocytes: a means to study maternal effect genes. *Genesis* **26**, 110-112 (2000).

6 Truett, G. E. *et al.* Preparation of PCR-quality mouse genomic DNA with hot sodium hydroxide and tris (HotSHOT). *Biotechniques* **29**, 52, 54 (2000).

7 Gal, A., Lin, P. C., Barger, A. M., MacNeill, A. L. & Ko, C. Vaginal fold histology reduces the variability introduced by vaginal exfoliative cytology in the classification of mouse estrous cycle stages. *Toxicol Pathol* **42**, 1212-1220, doi:10.1177/0192623314526321 (2014).

8 Schmittgen, T. D. & Livak, K. J. Analyzing real-time PCR data by the comparative C(T) method. *Nat Protoc* **3**, 1101-1108 (2008).

9 Chakravarthy, U., Douglas, A. J., Bailie, J. R., McKibben, B. & Archer, D. B. Immunoreactive endothelin distribution in ocular tissues. *Invest Ophthalmol Vis Sci* **35**, 2448-2454 (1994).

10 Choi, D. H. *et al.* Expression pattern of endothelin system components and localization of smooth muscle cells in the human pre-ovulatory follicle. *Hum Reprod* **26**, 1171-1180, doi:10.1093/humrep/der066 (2011).

11 Adur, J., Takizawa, S., Uchide, T., Casco, V. & Saida, K. High doses of ultraviolet-C irradiation increases vasoactive intestinal contractor/endothelin-2 expression in keratinocytes of the newborn mouse epidermis. *Peptides* **28**, 1083-1094, doi:10.1016/j.peptides.2007.03.009 (2007).

12 Takizawa, S. *et al.* Differential expression of endothelin-2 along the mouse intestinal tract. *J Mol Endocrinol* **35**, 201-209, doi:10.1677/jme.1.01787 (2005).

13 Binz, N. *et al.* Effect of vascular endothelial growth factor upregulation on retinal gene expression in the Kimba mouse. *Clin Exp Ophthalmol* **41**, 251-262, doi:10.1111/j.1442-9071.2012.02845.x (2013).
